# Uncovering Phenotypic Diversity and DArTseq Marker Loci Associated with Antioxidant Activity in Common Bean

**DOI:** 10.3390/genes11010036

**Published:** 2019-12-28

**Authors:** Muhammad Azhar Nadeem, Müttalip Gündoğdu, Sezai Ercişli, Tolga Karaköy, Onur Saracoğlu, Ephrem Habyarimana, Xiao Lin, Ruştu Hatipoğlu, Muhammad Amjad Nawaz, Muhammad Sameeullah, Fiaz Ahmad, Bok-Mi Jung, Gyuhwa Chung, Faheem Shehzad Baloch

**Affiliations:** 1 Department of Field Crops, Faculty of Agricultural and Natural Science, Bolu Abant Izzet Baysal University, Bolu 14030, Turkey; azharjoiya22@gmail.com (M.A.N.); sameepbg@gmail.com (M.S.); 2 Department of Horticulture, Faculty of Agriculture and Natural Sciences, Bolu Abant Izzet Baysal University, Bolu 14000, Turkey; gundogdumuttalip@gmail.com; 3 Department of Horticulture, Faculty of Agriculture, Ataturk University, Erzurum 25240, Turkey; sercisli@gmail.com; 4 Faculty of Agricultural Sciences and Technologies, Sivas University of Science and Technology, Sivas 58140, Turkey; tolgakarakoy73@hotmail.com; 5 Department of Horticulture, Faculty of Agricultural, Tokat Gaziosmanpasa University, Tokat 60010, Turkey; onur.saracoglu@gop.edu.tr; 6 CREA Research Center for Cereal and Industrial Crops, 40128 Bologna, Italy; ephrem.habyarimana@crea.gov.it; 7 School of life Sciences, The Chinese university of Hong Kong, Shatin, 999077, N.T., Hong Kong, China; alanlamsiu@gmail.com; 8 Department of Field Crops, Faculty of Agricultural, University of Cukurova, Adana 1000, Turkey; rhatip@mail.cu.edu.tr; 9 Education and Scientific Center of Nanotechnology, Far Eastern Federal University, Vladivostok 690950, Russian; amjad_ucauos@yahoo.com; 10 Jamil-ur-Rahman Center for Genome Research, International Center for Chemical and Biological Sciences, University of Karachi, Karachi 75270, Pakistan; fiazbiotechnologist@gmail.com; 11 Division of Food and Nutrition, Chonnam National University, Gwangju 61186, Korea; jbm@chonnam.ac.kr; 12 Department of Biotechnology, Chonnam National University, Chonnam 59626, Korea

**Keywords:** *Phaseolus vulgaris*, germplasm characterization, GWAS, genetic basis, genotype by environment interaction, mixed linear model

## Abstract

Antioxidants play an important role in animal and plant life owing to their involvement in complex metabolic and signaling mechanisms, hence uncovering the genetic basis associated with antioxidant activity is very important for the development of improved varieties. Here, a total of 182 common bean (*Phaseolus vulgaris*) landraces and six commercial cultivars collected from 19 provinces of Turkey were evaluated for seed antioxidant activity under four environments and two locations. Antioxidant activity was measured using ABTS radical scavenging capacity and mean antioxidant activity in common bean landraces was 20.03 µmol TE/g. Analysis of variance reflected that genotype by environment interaction was statistically non-significant and heritability analysis showed higher heritability of antioxidant activity. Variations in seed color were observed, and a higher antioxidant activity was present in seeds having colored seed as compared to those having white seeds. A negative correlation was found between white-colored seeds and antioxidant activity. A total of 7900 DArTseq markers were used to explore the population structure that grouped the studied germplasm into two sub-populations on the basis of their geographical origins and trolox equivalent antioxidant capacity contents. Mean linkage disequilibrium (LD) was 54%, and mean LD decay was 1.15 Mb. Mixed linear model i.e., the Q + K model demonstrated that four DArTseq markers had significant association (*p* < 0.01) for antioxidant activity. Three of these markers were present on chromosome Pv07, while the fourth marker was located on chromosome Pv03. Among the identified markers, DArT-3369938 marker showed maximum (14.61%) variation. A total of four putative candidate genes were predicted from sequences reflecting homology to identified DArTseq markers. This is a pioneering study involving the identification of association for antioxidant activity in common bean seeds. We envisage that this study will be very helpful for global common bean breeding community in order to develop cultivars with higher antioxidant activity.

## 1. Introduction

The world is facing a great threat of climate change, and extreme events throughout the world are being observed. Besides climate change, rapid growth in the world population is a global issue, and concerns have been raised to produce a sufficient quantity of food in terms of calories and nutrients [1]. A report issued by the food and agriculture organization (FAO) [2] stated that the daily average per capita calories available to the world population were 2789 kcal in the year 2000, but it is estimated that 3130 kcal will be required on a daily basis during the year 2050. In this context, grain legumes are key commodities having great potential to mitigate world food shortages and provide well balanced and cheap food [3]. 

The common bean (*Phaseolus vulgaris* L.) is one of the most important proteinaceous crops of Latin America. The common bean serves a source of food for more than 300 million people [4]. Although the common bean is an underrated crop, the presence of higher quantity of proteins, carbohydrates, vitamins, and minerals makes it a rational food of choice, particularly for poor people all around the world [5]. Global common bean production was 23 million tons in 2010 and 26 million tons in 2016 [6]. The common bean reflects a great level of genotypic and phenotypic diversity, and domestication of this crop resulted in two distinct gene pools—the Mesoamerican gene pool from Central America and the Andean gene pool from the Andes Mountains in South America [7]. Turkey is not the center of origin of the common bean, and it is known that this crop was introduced to Turkey from Europe in the 17th century [8]. However, hundreds of landraces, especially those having bigger seed size due to local consumer preference, are found in Turkey. The common bean is a very popular food commodity for Turkish people and is an important part of their daily diet [9]. An increase in the yield of the common bean has been observed in Turkey during the last two decades; 212,758 tons were produced in the year 2010, and 651,094 tons in the year 2016 [6], making up a share of about 2.75% of the world production. To date, 200 fresh bean and 39 dry bean cultivars have been registered in Turkey (Variety Registration and Seed Certification Center; www.tarimorman.gov.tr). According to Ozturk et al. [10], the observed gradual rise in common bean production is due to the development of improved cultivars reflecting better adaptation to environmental stresses. 

Advancements in the molecular markers and sequencing technologies have boosted breeding activities [11]. Among major techniques, genome-wide association studies (GWAS) and quantitative trait loci (QTL) mapping are the two main approaches used to elucidate the genetic basis for traits of interest. QTL mapping or bi-parental mapping is mainly used for the investigation of QTLs associated with traits of interest. However, in addition to its advantages, QTL mapping also has several drawbacks, like low recombination, being time-consuming, and the population specificity of the identified QTLs [11]. GWAS overcomes all the drawbacks of QTL mapping, and the identified markers through this approach can be used for any population [12]. GWAS has been successfully carried out for the common bean in order to elucidate marker-trait association for agronomic traits [13], seed weight [14], disease resistance [15], cooking properties [16], anthracnose resistance [17], and drought tolerance [18]. These studies resulted in the identification of some candidate genes that can be helpful in the development of new genetic stocks for breeding perspectives in the common bean. 

Antioxidants are an important class of chemicals present in our food and play an essential role in reducing the oxidative stress of the physiological system [19]. Antioxidants play a significant role against various disorders like cancer, aging, cardiovascular disease, cataracts, immune system decline, and brain dysfunction. Antioxidants minimize the chances of these diseases by preventing the production of free radicals [20]. Sudhir et al. [21] stated that higher antioxidants in food minimize the incidence of cardio and cerebrovascular diseases. Oxygen under various conditions may have serious deleterious effects on the body [22]. Oxygen is a highly reactive atom with the ability to become part of potentially damaging molecules known as “free radicals.” These free radicals have the ability to attack healthy body cells, leading to various diseases like cardiovascular disease, cancer, brain dysfunction, and a decline in the immune system [20,21,22,23]. Antioxidants can electrons to stabilize these free radicals and prevent their detrimental effects. Antioxidants protect the body from both endogenous and exogenous molecules [24]. Sikora et al. [25] stated that food containing a higher concentration of antioxidants can significantly affect the increase of the reactive antioxidant potential of the organism and decrease the risk of some diseases caused by the production of free radicals. Antioxidants can also be endogenous or exogenous; exogenous antioxidants enter the body when we eat antioxidant-rich food, while endogenous antioxidants are solely produced by the body. Exogenous antioxidants are found in plant and animal sources, and those of plant origin are of great interest because of their presence in relatively higher quantities [26,27].

The role of antioxidants against various stress conditions in plants has been well documented [28]. Plant species containing a higher level of antioxidant contents, either constitutive or induced, reflect a higher resistance to environmental stresses [29]. According to Silvana et al. [30], the extent of oxidative damage to plant cells when exposed to abiotic stresses is mainly governed by the capacity of their antioxidant defense system. As antioxidant activity is a key trait, its manipulation can be a relevant target for common bean breeding community to develop new varieties having improved antioxidant activity. This in turn will be helpful for improving human health. GWAS has been used previously for the identification of genes associated with antioxidant activity and total flavonoid contents in barley [31] and rice [32] in order to investigate polyphenol contents and antioxidant activity. Despite a good understanding of the role of antioxidants for human health, the genetic basis for antioxidant activity has not been elucidated for most of crops. The common bean is a good source of antioxidants [29], and Cardador-Martiänez et al. [33] stated that bean extract from the hull contains a significant level of antioxidants. Weidner et al. [34] explored the antioxidant levels in different colored common bean genotypes and observed a good amount of antioxidant capacity. The common bean serves as a source of food for a large population of the world, so variations in the common bean for antioxidant activities can be used to develop cultivars with better antioxidant content. Therefore, an effort is made through this study to explore the variations for antioxidant activity and antioxidant activity–associated markers in Turkish common bean germplasm.

## 2. Materials and Methods

### 2.1. Plant Material

**Figure 1:**
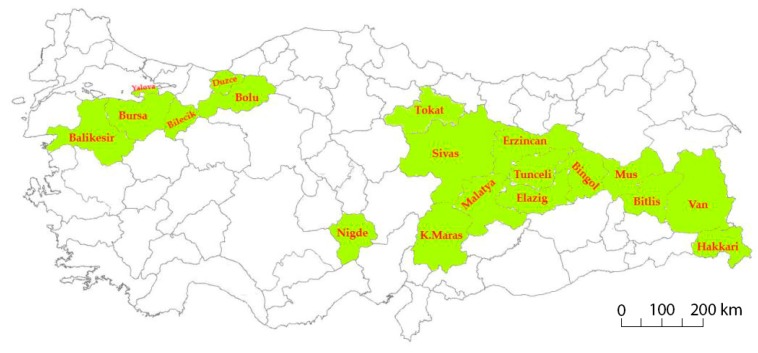
Collection provinces of Turkish common bean germplasm.

A total of 182 common bean landraces and six commercial cultivars (Akman, Goynuk, Karacaşehir, Onceler, Goksun, and Akdag) were used as plant material in this study. Germplasm was collected from 19 geographical regions of Turkey (Table S1; Figure 1) and maintained at Bolu Abant Izzet Baysal University (BAIBU). Commercial cultivars used in this study were developed through a single plant selection and have been used as standard cultivars in previous studies [35,36].

### 2.2. Field Experiment and Phenotypic Evaluation

Field experiments were conducted during 2017 and 2018 at two experimental sites—BAIBU (40°44′45″ N/31°37′44″ E, altitude; 752 m) and Cumhuriyet University Sivas (39°42′16.69″ K/37°01′57.48″ D, altitude; 1293 m). The experiment was laid out according to augmented block design, which has been found to be more effective, precise, time-saving, and trustworthy compared to other experimental designs [37]. Augmented block design contains more than one control cultivar that are taken as replicated treatments, and these cultivars are repeated in each block. These control cultivars are used to standardize the data to calculate adjusted means. Evaluated means are later used for various statistical analyses [37,38]. In this study, seeds were planted in elementary plots, each consisting of a 2 m row with a 50 cm inter-row and 10 cm intra-row spacing. Studied germplasm was sown according to augmented block design in eight blocks. Six commercial cultivars (Akman, Goynuk, Karacaşehir, Onceler, Goksun, and Akdag) were included in each block as a control group. Sowing was done on 27 April 2017 and 16 April 2018, respectivelyat the BAIBU, while the trial in Sivas location was sown on 15 May 2017 and 19 May 32018. Di-ammonium phosphate (DAP) (130 kg/ha) and ammonium sulfate (51 kg/ha) were used as a source of fertilizer, while standard agriculture practices were performed at both locations. Harvesting was done at the time of 90% pods per plant maturity.

### 2.3. DNA Extraction and Genotyping for DArTseq Markers

DNA was extracted from two-week-old seedlings according to CTAB protocol of Doyle and Doyle [39] with some modifications [40]. Agarose gel electrophoresis (0.8%) was used to check the purity of isolated DNA and quantification was performed through DS-11 FX series spectrophotometer/fluorometer (Denovix, Wilmington, DE, USA). High-quality DNA was further diluted and a final concentration of 50 ng μL-1 was prepared. For genotyping-by-sequencing (GBS), the prepared DNA samples were processed at Diversity Array Technology Pty, Ltd., Bruce, Australia (http://www.diversityarrays.com/). DArTseq analysis was carried out as previously described [9].

### 2.4. Extraction and Determination of Total Antioxidant Activity

Antioxidant activity was measured using ABTS radical scavenging capacity. For the investigation of antioxidant activity, TEAC extract was firstly prepared to calculate trolox equivalent antioxidant capacity (TEAC). For this purpose, dry samples were extracted with acetone, water, and acetic acid solution (70/29.5/0.5), respectively for ten days. For TEAC analysis, 7 mM ABTS (2,2′-Azino-bis 3-ethylbenzothiazoline-6-sulfonic acid) was mixed with 2.45 mM potassium sulfate for 12–16 h as suggested by Saracoglu et al. [41]. This solution was then mixed with 20 mM sodium acetate (pH 4.5) buffer solution. Finally, 30 μL of extract was mixed with 2.97 mL of prepared buffer and incubated for 10 min and measured on a spectrophotometer at a wavelength of 734 nm to 0.700 ± 0.01. Absorbance values obtained were calculated with Trolox (10–100 µmol/L) and presented as µmol TE/g.

### 2.5. Statistical Analysis

#### 2.5.1. Phenotypic Data Analysis

An online software for augmented block design developed by Rathore et al. [42] was used to derive statistical inferences. The analysis was performed in two steps. First, analysis of variance (ANOVA) was investigated within the environments, and adjusted means were derived. Second, these evaluated means were used to run ANOVA across the environments, solving the appropriate mixed model equation to contemporarily account for both genotype and genotype × environment interaction effects. The Fligner–Killeen test was also computed before combined analysis to evaluate the homogeneity of variance. Environmental effects were taken as random, and genotypic effects were taken fixed in the computed mixed model equation, as suggested by Gomez and Gomez [43]. For the calculation of heritability, appropriate variance components were derived from the linear mixed model equation fitted attributing genotype and environment random effects as reported by Habyarimana [44]. The model was fitted with a restricted maximum likelihood using the R software [45]. The boxplot and frequency distribution of antioxidant activity were investigated using GenStat software [46]. Pearson’s correlation coefficient among various colored seeds and antioxidant activity was investigated using the statistical software XLSTAT (www.xlstat.com).

#### 2.5.2. Population Structure and GWAS Analysis

Population structure was carried as described by Nadeem et al. [9]. A mixed linear model (MLM, Q + K) approach was used to investigate the marker-trait association. Q-metrics (Q) and kinship (K) were used to correct the population and family structure during association analysis. The kinship matrix was investigated by following the methodology reported by Bradbury et al. [47] through TASSEL 5.0.5 (https://tassel.bitbucket.io) software. Linkage disequilibrium (LD) was estimated for all DArTseq markers with *r*^2^ based on allele frequencies [48,49]. Pairwise LD values of polymorphic sites were plotted on both the X- and Y-axis to generate LD heat map. To calculate LD decay, the pairwise correlations among linked markers in significant LD (*p* < 0.001) were used. The threshold for LD decay was considered below *r*^2^ = 0.1. Results of association analysis reflected that *p* value (marker) shows whether a marker is associated with a trait, and *R*^2^ (marker) defines the proportion of phenotypic variation accounted for by a significant marker [49,50]. Both Bonferroni and FDR thresholds were used, while DArTseq markers having FDR and Bonferroni *p* = 0.01 thresholds were taken significantly associated with antioxidant activity. Statistically significant markers for antioxidant activity were visualized through the Manhattan plot in R 3.4.1 statistical software (http://www.r-project.org/) by using qq-man R Package [51]. Quantile-Quantile (q-q) plot was drawn to visualized important *p*-value distributions (expected vs. observed *p*-values on a –log10 scale) through TASSEL 5.2.50 (https://tassel.bitbucket.io) software.

#### 2.5.3. Putative Candidate Gene Analysis

Sequences of DArTseq markers associated with the antioxidant activity were used to BLAST-search against common bean genome in Phytozome V.12.1 (http://phytozome.jgi.doe.gov/pz/portal.html), the legume information system (LIS: https://legumeinfo.org/), and the National Center for Biotechnology Information (NCBI) (http://www.ncbi.nlm.nih.gov/) databases to detect putative genes homologous to these sequences.

## 3. Results

### 3.1. Phenotypic Diversity Evaluation

**Figure 2:**
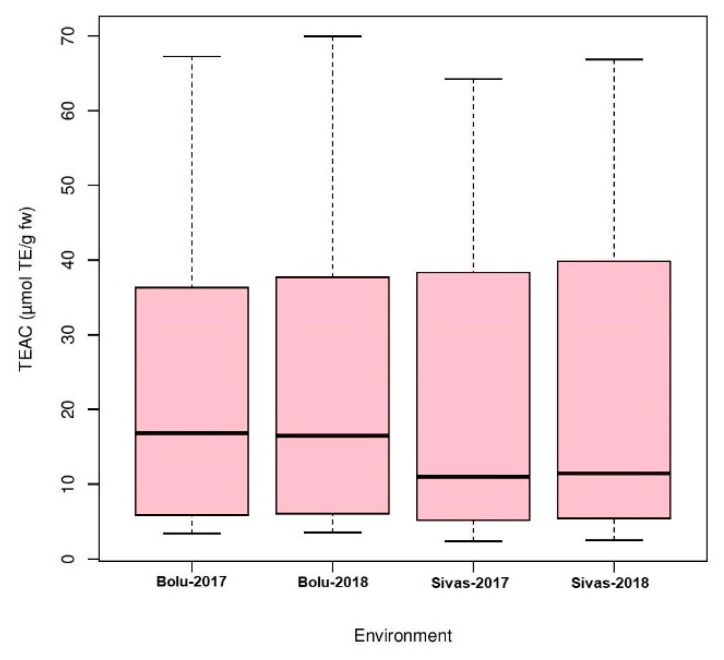
Boxplot analysis of antioxidant activity in Turkish common bean germplasm under four environments.

**Figure 3:**
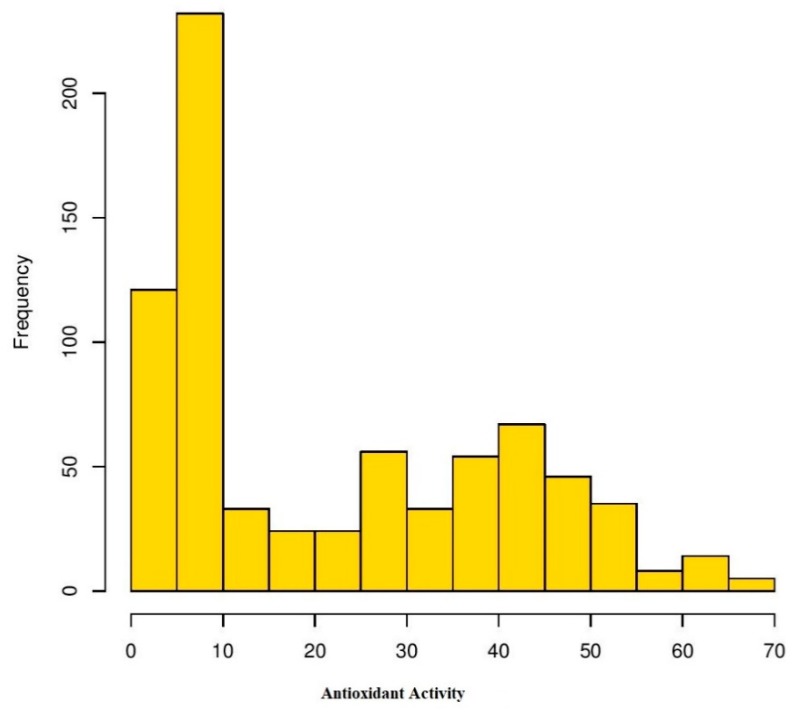
Frequency distribution of antioxidant activity in Turkish common bean germplasm under four environments.

Analysis of variance (ANOVA) was performed for four environments to understand the effects of genotype, environment, and the interaction of both (GEI) for antioxidant activity in Turkish common bean germplasm (Table 1). Genotypic effects were found to be statistically significant at (*p* < 0.05; F value 11.9), while GEI was statistically non-significant for antioxidants activity in this study. Heritability analysis revealed the existence of higher heritability (0.92) (Table 1). Antioxidant activity was evaluated as TEAC for each environment for both locations (Table S2). At BAIBU in 2017, the mean TEAC for common bean landraces was 19.78 ranging 1.8 (Hakkari-65) to 67.2 (Balikesir-19) (Table S3). At the same location in 2018, 20.25 was the mean TEAC, while 1.9 (Hakkari-65) and 69.9 (Balikesir-19) were the minimum and maximum TEAC, respectively. At the Sivas location in 2017, mean TEAC was 19.65, while minimum and maximum TEAC reflected by Hakkari-65 and Bitlis-22 landraces were 2.4 and 64.3, respectively. Mean TEAC was 20.43 at Sivas location during 2018, while minimum and maximum TEAC reflected by Hakkari-65 and Bitlis-22 landraces were 2.5 and 66.8, respectively. Overall mean TEAC in Turkish common bean landraces was 20.03, ranging from 32.15 (Hakkari-65) to 60.48 (Balikesir-19). Mean TEAC in common bean cultivars at Bolu in 2017 and 2018 were 13.9 and 14.3, respectively (Table S3). Mean TEAC in common bean cultivars at Sivas in 2017 and 2018 were 15.7 and 16.3, respectively, while overall mean TEAC in common bean cultivars during the whole period of study was 15.1. Boxplot analysis also confirmed the results of ANOVA by revealing that there are no significant environmental effects on the seed antioxidant contents (Figure 2). Frequency distributions for TEAC in common bean are presented in Figure 3, which shows that most of the landraces have less than 10 µmol TE/g TEAC. Mean antioxidant activity in the colored seeds and white seeds were 30.04 and 16.22 µmol TE/g TEAC, respectively. Correlation analysis between antioxidant activity and seed caot colors was also performed, which revealed a negative correlation of antioxidant activity with white seeds (Table 2). 

### 3.2. Population Structure and LD

Whole-genome DArTseq profiling of Turkish common bean germplasm resulted in a total of 15,608 DArTseq markers. This marker dataset was filtered to retain 7900 high-quality markers with less than 5% missing data, PIC value > 0.10, call rate > 0.60, and 100% reproducibility. The Bayesian clustering model implemented in STRUCTURE software grouped studied germplasm into two groups; population A (red) containing 117 landraces (59.57%) and population B (green) 71 landraces (37.76%) (Figure S1). The 7900 highly informative DArTseq markers were used for LD and GWAS analysis. Based on each chromosome, the number of mapped markers ranged from 931 (Chr. 2) to 591 (Chr. 6) (Table 3). The mean distance between markers on whole-genome was 15.54 Mb with a range of 13.15 (Chr 7) to 18.99 Mbps (Chr. 2). Mean *r*^2^ was 0.47, and 54% was the mean significant LD (Table 3). Mean LD decay for whole-genome was 1.15 Mbps which ranged from 1.05 Mbps for chromosome 9 to 1.3 for chromosome 5. Nonlinear trend line of LD measure *r*^2^ vs. the physical map distance decayed with almost 1.3 Mbs to *r*^2^ value of 0.5 (Figure S2). Linkage disequilibrium measured *r*^2^ plotted vs. *p*-value between pairs of DArTseq markers revealed LD decay on LG6 (Figure S3).

### 3.3. Marker Trait Association for Antioxidant Activity

**Figure 4:**
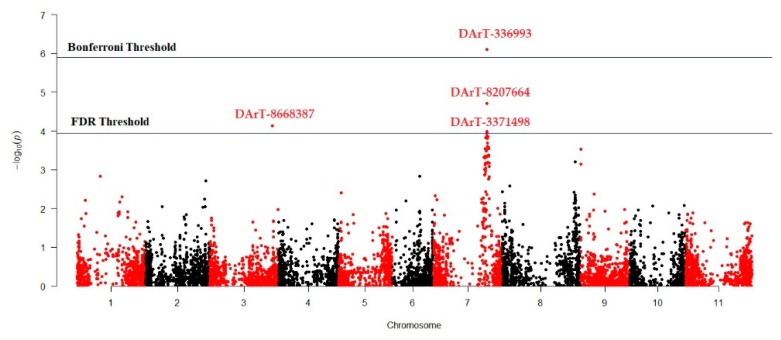
Manhattan plot reflecting marker trait association for antioxidant activity in common bean germplasm.

**Figure 5:**
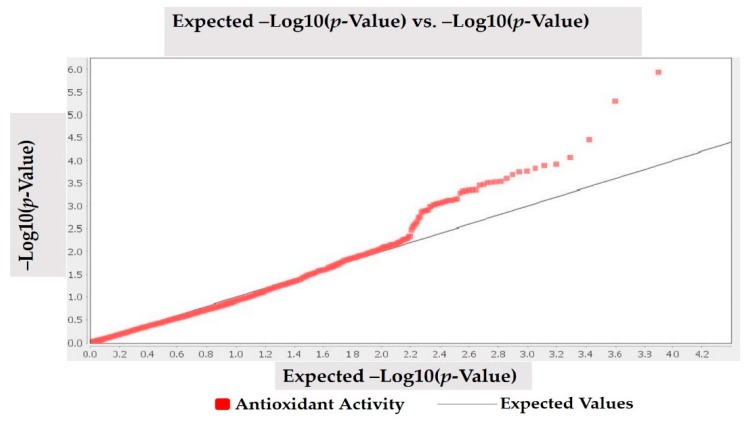
Quantile-quantile (q-q) plots for antioxidant activity in Turkish common bean germplasm.

MLM (Q + K) model was applied to investigate marker-trait association for antioxidant activity in studied Turkish common bean germplasm. MLM based on Q + K model showed that out of 7900 DArTseq markers, only four markers (DArT-3369938, DArT-8668387, DArT-8207664, and DArT-3371498) reflected statistically significant association for antioxidant activity (Table 4; Figure 4). The phenotypic variation percentage (R^2^) reflected by each marker ranged between 0.094% to 0.156% for DArT-3371498 and DArT-3369938, respectively. The *p*-value of each marker reflecting significant level of association between marker and antioxidant activity is presented in the form of Manhattan plots (Figure 4). Manhattan plots showed that markers associated with antioxidant activity are present on chromosomes 3 and 7. Quantile-quantile (q-q) plot (Figure 5) reflected the goodness of fit of the best model by implementing *p*-value distributions (expected vs. observed *p*-values on a -log10 scale).

### 3.4. Putative Candidate Genes for Antioxidant Activity

Sequences of all four identified linked markers were used as queries to BLAST-search against Phytozome (V.12.1), LIS, and NCBI databases to detect predicted genes homologous to these sequences. A zinc-finger protein family gene (Phvul.009G180200.2) located on Ch Pv07 was retrieved in BLAST using DArT-3369938 marker as a querry (Table 4). The search for the homologous candidate gene against DArT-8207664 marker resulted in the retrieval of Phvul.007G281200.1 on chromosome Pv07. The gene encodes a methylene tetrahydrofolate reductase also known as the MTHFR gene, and folic acid production is predicted function of this gene. A search against the query marker DArT-8668387 resulted in the retrieval of Phvul.003G239700 located on chromosome Pv03. This gene encodes for a UDP-glycosyltransferase superfamily protein. Phvul.007G171700.1 was found against DArT-3371498. The gene located only 20 kb upstream of DArT-3371498 belongs to the Pentatricopeptide repeat family (PPR_2).

## 4. Discussion

In this study, a total of 182 Turkish common bean landraces and six commercial cultivars were used to identify marker trait association for antioxidant activity. Results of phenotypic characterization for antioxidant activity showed a great level of diversity ranging from 2.15 to 60.48 with the mean TEAC of 20.03 µmol TE/g (Table S3). The observed range and average TEAC in the studied germplasm was much higher than reported by Huber et al. [52], Weidner et al. [34], and Bojilov et al. [53]. Antioxidant activity during 2018 at both locations was recorded as higher compared to 2017. García-Díaz et al. [54] also observed higher antioxidant activity in common bean seeds in one year as compared to the other year of study. These differences in the antioxidant activity of common seeds grown in different years have been previously explained to be influenced by environmental variations, crop management practices, and genetic differences [55]. Frequency distribution reflected that a good number of landraces acquired TEAC contents similar to the results of earlier studies [34,35,36,37,38,39,40,41,42,43,44,45,46,47,48,49,50,51,52,53]. The ANOVA was performed within and across the four environments to evaluate the genotypic effect and explore the GEI for antioxidant capacity. The ANOVA confirmed that statistically significant variations in TEAC are due to the genetic background of landraces, while GEI reflected a non-significant effect for antioxidant capacity. These results were further strengthened by the results of heritability analysis. Higher heritability (0.92) in this study reflected that this trait is mainly under the control of genetic forces, while the environmental forces have very little effect. García-Díaz et al. [54] investigated the effect of cropping season and genotype on antioxidant activity in the common bean and revealed that variations are mainly due to genotypic effect. Di Silvestro et al. [56] investigated how genotype and environment effect antioxidant activity in wheat under multiyear experiments. A lesser effect of the environmental forces was observed in their results supports our observations of the common bean. A similar study on corn concluded that most of the variations for studied traits are contributed by the genotypes as compared to environmental forces [57].

We also observed variations in seed color and recorded that white color (58% of the accessions) was prevalent in our selected population. Seed color in the common bean is associated with bioactive compounds, such as anthocyanin and tannins which are found in higher quantity in colored seeds as compared to white colored seeds [58]. Earlier studies explored the relationship between the seed color and antioxidant activity in common bean and concluded the role of seed color in higher antioxidant activity [58,59]. In this study, mean antioxidant activity was found much higher in colored seeds as compared to the white ones. These results are in line with the previous studies stating the existence of higher antioxidant activity in colored seeds as compared to white seeds [58,59,60]. Pearson’s correlation coefficient showed a highly significant correlation between colored seeds (brown, beige) and antioxidant activity. On the other hand, there was a negative correlation between white seeds and antioxidant activity. Earlier studies revealed the presence of higher anthocyanin in the seed coat of the colored seed, which ultimately contributes toward a higher antioxidant activity [58,59].

### 4.1. DArTseq Markers Analysis and LD

A total of 7900 markers were employed in this study to investigate population structure and marker-trait association for antioxidant activity. The number of mapped markers ranged from 931 (Chr. 2) to 591 (Chr. 6) (Table 3). The mean distance between markers on the whole nuclear genome was 15.54 Mb with a range of 13.15 (Chr. 7) to 18.99 Mbps (Chr. 2). The number of markers used in this study was much higher than earlier studies used for GWAS in various plant [61,62], which in turn leads toward a more efficient genome-wide screening. Using marker information, the structure algorithm divided whole germplasm into two heterotic populations on the basis of their 100-seeds weight, TEAC contents, and collection point. Population A clustered a total 117 landraces, while 71 landraces clustered in population B. Akdag, Onceler, and Goynuk cultivars grouped in population A and Akman, Goksun, and Karacasehir reflected much similarity with population B. Landraces clustered in population A mainly contained higher 100-seed weight and relatively more TEAC contents, while landraces having lower to moderate TEAC contents with lower 100-seed weight clustered into population B. Geographic locations also played a role in the clustering and landraces from same provinces were grouped together. Earlier studies have confirmed the clustering of common bean germplasm based on plant height, 100-seed weight, and collection point. These previous reports strengthen the findings of our study [63]. 

Linkage disequilibrium is a nonrandom association of alleles at particular loci in a sampled population genome [64]. In this study, mean *r*^2^ was 0.47 while the mean significant LD was 54% (Table 3). The maximum and minimum LD was 59.8% and 45.89% reflected by chromosomes 11 and 9, respectively. Mean LD decay was 1.15 Mb for the whole genome and ranged from 1.05 Mbps for chromosome 7 to 1.3 Mbps for chromosome 5. A nonlinear trend line between *r*^2^ vs. distance bp showed that LD decayed at 1.3 Mbs and *r*^2^ value was 0.5 (Figure S2). When an LD decay plot is constructed, usually the distance point is looked for where the LD value (*r*^2^) decreases below 0.1 or half of D’ strength (D’ = 0.5) based on curve of nonlinear logarithmic trend line [65,66]. This shows a rough estimation of extent of LD, however, highly significant threshold LD values (*r*^2^ ≥ 0.2) are used for more precise LD calculation. Our results are in a disagreement with the findings of Campa et al. [67], who stated that chromosome 9 shows maximum LD. Linkage disequilibrium measured *r*^2^ plotted vs. *p*-value between pairs of DArTseq markers the studied germplasm showed a relatively bigger LD decay on LG6 (Figure S3). Mean *r*^2^ found in this study was slightly lower as compared to the findings by Ates et al. [68]. The higher LD in their study may be due to human selection, which leads in particular usage, combining distinct variations through a long history of cultivation and multiple nationwide expeditions.

### 4.2. Marker Trait Association and Putative Candidate Genes for Antioxidant Activity

We used GWAS for the whole-genome screening of the selected germplasm. This approach has rapidly gained the attention of breeders to identify genomic regions for various traits of interest that can be used for marker-assisted breeding [69]. For the investigation of marker–trait association, the MLM (Q + K) approach was used during GWAS analysis to eliminate possible spurious associations [68]. In this study a total of four markers (DArT-3369938, DArT-8668387, DArT-8207664, and DArT-3371498) were significantly associated with seed antioxidant contents (Table 4; Figure 4). DArT-3371498 accounted for 15.6% of the total variation and was the most statistically significant marker, while DArT-3371498 accounted for the least variation among these four identified markers. The Manhattan plot showed that DArT-3369938, DArT-8207664, and DArT-3371498 are located on chromosome 7, while DArT-8668387 reflected association with chromosome 3. As these markers reflected significant association for antioxidant activity in the common bean, these markers can be used for marker-assisted breeding to develop cultivars with better antioxidant activity. 

Located only 100 kb upstream of DArT-3369938, on Pv09, *Phvul.009G180200.2* is putative candidate gene to explain the phenotypic variation associated with this marker. This gene encodes for zinc-finger domain–containing protein. Zinc-finger proteins (ZFPs) constitute large protein families and have an important role in various plant developmental stages and to biotic and abiotic stress [70]. Hichri et al. [71] stated that this protein family contains transcription factors that control various aspects of plant development and shown a pivotal role in abiotic stress tolerance. Cao et al. [72] found that ZFPs have resistant to biotic stress such as rice blast fungus infection. Cheuk and Houde [73] explored the role of ZFPs in abiotic stress and investigated 53 Q-type C2H2 zinc-finger proteins (TaZFPs) from *Triticum aestivum*, and stated that these TaZFPs have positive response to high light (44/53), H_2_O_2_ (37/53), drought (37/53), and flooding (31/53). Zang et al. [74] confirmed that this protein is involved in abiotic stress by investigating abiotic stress in Arabidopsis and concluded that ZFPs increase the salt and osmotic tolerance in this plant through a series of physiological processes. Zhang et al. [75] identified ZFPs in rice and concluded that overexpression of ZFPs in their study elevated the activities of antioxidant enzymes and enhanced the tolerance of rice plants against water and oxidative stresses.

The BLAST search against DArT-8668387 retrieved a gene (*Phvul.003G239700*) encoding UDP-Glycosyltransferase superfamily protein; a multifunctional protein family in plants. Glycosylation is one of the final steps involved in the biosynthesis of triterpenoid for the production of various plant defensive compounds like phenolic, glucosinolates, salicylates, and anthocyanins [76]. UDP-glycosyltransferases (UGTs) are one of the biggest gene families in the plant kingdom and play an important role in transferring sugar, controlling various metabolic processes, and play an effective role against various biotic and abiotic stresses [77,78]. Various studies have explored the biological role of the putative UGTs against different abiotic stresses. Li et al. [77] comprehensively explained the role and functioning of a homolog of this protein in enhanced plant tolerance to low temperatures, drought, and salt stresses in Arabidopsis. Sun et al. [79] found that ectopic expression of *UGT85A5* in tobacco resulted in enhanced salt stress tolerance. Rehman et al. [78] performed a comparative genomic and transcriptomic analyses study for brassica species and Arabidopsis and found the role of UDP-Glycosyltransferase in biotic and abiotic stresses. 

*Phvul.007G281200.1* resulted as the putative candidate gene for DArT-8207664 marker that encodes for the methylene tetrahydrofolate reductase MTHFR gene. MTHFR enzyme is involved in the metabolism of folate and homocysteine (Hcy). This gene is linked with folate metabolism which plays a positive effects on oxidative stress by its protecting action against cell death and increased folic concentrations stabilize MTHFR enzyme [80]. Earlier studies have found the synergetic effect of folic acid on growth, yield, and yield quality of many plant species. Folic acid can play an effective role by catching the free radicals or active oxygen produced during the stress conditions and help the plant defense against environmental stresses [81]. *Phvul.007G171700.1* encodes for PPR containing plant protein (RNA-binding proteins) having a key role in post-transcription via RNA editing, cleavage, splicing, stability, or translation in plastids or mitochondria [82]. Earlier studies revealed that PPR proteins might be target genes of some miRNAs regulating abiotic stress responses [83]. Zsigmond et al. [84] revealed that PPR proteins are involved in oxidative respiration that contributes to abiotic stress tolerance in Arabidopsis. Recently, Chen et al. [85] aimed to investigate the functioning of PPR gene family in rice and explored the role of this family under stress condition. They investigated the expression pattern of this gene family and concluded that PPR proteins have crucial roles in response to different abiotic stresses in rice.

As is obvious from the previously discussed evidence, our diverse association panel reflected a wide range of antioxidant activity in common bean seeds. These genetic variations can be utilized for various common bean improvement programs. The role of antioxidants in human health has been universally accepted, and earlier studies revealed that higher antioxidant levels in food can prevent various disorders [20,21,22]. Germplasm characterization is an important way to elucidate novel variations which can be used for the breeding perspective of any crop [86,87,88]. Therefore, there is a need to identify the genetic basis for association with antioxidant activity. The common bean serves as a source of food for millions of people all over the world, and thus, the intake of beans with higher antioxidant levels will reduce the risk of various disorders. We identified four DArTseq markers (DArT-3369938, DArT-8668387, DArT-8207664, and DArT-3371498) with significant association for antioxidant activity in Turkish common bean germplasm. These DArTseq marker can be used for the marker-assisted breeding of the common bean in order to develop cultivars having improved antioxidant activity. 

## 5. Conclusions

A wide range of phenotypic variations were observed for antioxidant activity in Turkish common bean germplasm. Heritability analysis revealed that antioxidant activity is controlled by genotypic effect. A negative correlation was observed between antioxidant activity and white seeds. This is a pioneer study revealing the marker trait association for antioxidant activity in Turkish common bean seeds. A total of four DArTseq markers showed statistically significant association for seed antioxidant activity, and these markers can be used in the future for the development of common bean cultivars with better antioxidant contents. 

## Figures and Tables

**Table 1:** Summary of analysis of variance and estimates of heritability in Turkish common bean germplasm.

Variables	Df	Sum Sq	Mean Sq	F Value	Pr (>F)
LINE	187	199487	1066.8	11.937	<2 × 10^−16^
ENV	3	115	38.2	0.427	0.734
Residuals	561	50133	89.4		
Heritability	0.92				

**Table 2:** Pearson’s correlation coefficient between various seeds colors and antioxidant activity.

Variables	White	Purple	Brown	Beige	Yellow	Dark red	Black	Antioxidant
White	1	−0.448 **	−0.328 **	−0.427 **	−0.236 **	−0.253 **	−0.177	−0.422 **
Purple		1	−0.105	−0.137	−0.076	−0.081	−0.057	0.108
Brown			1	−0.100	−0.055	−0.059	−0.042	0.214 **
Beige				1	−0.072	−0.077	−0.054	0.309 **
Yellow					1	−0.043	−0.030	−0.014
Dark red						1	−0.032	0.060
Black							1	0.080
Antioxidant								1

Statistically significant ** (*p* < 0.01).

**Table 3:** Distribution and linkage disequilibrium of DArTseq markers on different chromosome of common bean.

Chr.	Number of Markers	Mean Distance (Mbs)	*r* ^2^	Significant LD (%)	LD Decay (Mb)
1	658	12.61	0.52	53.76	1.2
2	931	18.99	0.48	56.47	1.2
3	863	16.53	0.51	53.61	1.15
4	632	13.80	0.41	54.58	1.18
5	659	16.38	0.39	59.16	1.3
6	591	18.48	0.50	55.16	1.15
7	680	13.15	0.45	49.55	1.08
8	863	14.47	0.46	55.4	1.15
9	637	17.03	0.59	45.89	1.05
10	599	13.86	0.45	50.62	1.1
11	787	15.68	0.46	59.8	1.2
	7900	15.45	0.47	54.0	1.15

**Table 4:** Significant marker-trait associations identified for antioxidant activity in Turkish common bean germplasm.

Marker	Chromosome	Position	*p*-Value	R^2^	Putative Gene
DArT-3369938	7	40398124	7.93 × 10^−7^	0.15657	*Phvul.009G180200.2*
DArT-8668387	3	47385021	7.40 × 10^−5^	0.09497	*Phvul.003G239700*
DArT-8207664	7	40398185	1.93 × 10^−5^	0.12493	*Phvul.007G281200.1*
DArT-3371498	7	40409459	1.03 × 10^−4^	0.09404	*Phvul.007G171700.1*

## Supplementary Materials

The following are available online at https://www.mdpi.com/2073-4425/11/1/36/s1, Figure S1: Population structure of Turkish common bean germplasm revealed by DArTseq markers, Figure S2: Scatter plot reflecting association between linkage disequilibrium (*r*^2^) and distance bp, Figure S3: Linkage disequilibrium (LD) measured r2 plotted vs. P-value between pairs of DArTseq markers in Turkish common bean germplasm Table S1: Passport data of Turkish common bean germplasm used in this study, Table S2: Antioxidant activity (µmol TE/g fw) in four environments for Turkish common bean germplasm, Table S3: Comparison of mean, maximum and minimum for antioxidant activity (µmol TE/g fw) between Turkish common bean landraces and commercial cultivars under four environments.
