# Supplementary material for: Uncovering Phenotypic Diversity and DArTseq Marker Loci Associated with Antioxidant Activity in Common Bean
Source: Genes (Basel). 2019 Dec 28;11(1):36. doi: 10.3390/genes11010036 (PMC7016922; doi:10.3390/genes11010036)
Supplement: Supplementary file 1 [file genes-11-00036-s001.zip › Table S2.docx]

**Table S2.** Antioxidant activity (µmol TE/g fw) in four environments for Turkish common bean germplasm

| Landraces | Bolu 2017 | Bolu 2018 | Sivas 2017 | Sivas 2018 | Mean | Seed color |
| --- | --- | --- | --- | --- | --- | --- |
| Bingol-1 | 4.7 | 4.9 | 6.9 | 7.2 | 5.93 | White |
| Bingol-6 | 8.5 | 8.7 | 11 | 11.4 | 9.90 | White |
| Bingol-7 | 4.8 | 5 | 5.2 | 5.5 | 5.13 | White |
| Bingol-11 | 5.2 | 5.4 | 5.4 | 5.7 | 5.43 | White |
| Bingol-16 | 3.6 | 3.7 | 7 | 7.2 | 5.38 | White |
| Bingol-18 | 44.1 | 44.9 | 27.7 | 28.8 | 36.38 | Purple |
| Bingol-25 | 41.4 | 42.1 | 52.3 | 54.4 | 47.55 | Beige |
| Bingol-33 | 5.1 | 5.3 | 5.9 | 6.1 | 5.60 | White |
| Bingol-36 | 5.3 | 5.5 | 5.4 | 5.6 | 5.45 | White |
| Bingol-44 | 3.4 | 3.5 | 4.8 | 5 | 4.18 | White |
| Bingol-45 | 20.1 | 20.1 | 5.1 | 5.3 | 12.65 | White |
| Bingol-52 | 21.7 | 21.5 | 5.7 | 5.9 | 13.70 | White |
| Bingol-53 | 3.82 | 3.87 | 5.2 | 5.4 | 4.57 | White |
| Bingol-58 | 12.1 | 12.6 | 6.1 | 6.3 | 9.28 | White |
| Bingol-60 | 2.5 | 2.6 | 6.7 | 6.9 | 4.68 | White |
| Bingol-61 | 7.6 | 7.9 | 6.6 | 6.8 | 7.23 | White |
| Bingol-63 | 18.5 | 17.2 | 5.7 | 5.9 | 11.83 | White |
| Bingol-65 | 6.5 | 6.7 | 4.6 | 4.7 | 5.63 | White |
| Hakkari-7 | 14.9 | 13.5 | 5 | 5.2 | 9.65 | White |
| Hakkari-11 | 41.6 | 40.3 | 42.7 | 44.4 | 42.25 | White |
| Hakkari-12 | 41.9 | 43.6 | 47.1 | 48.9 | 45.38 | Brown |
| Hakkari-13 | 4.4 | 4.4 | 4.1 | 4.3 | 4.30 | White |
| Hakkari-16 | 20.1 | 20.9 | 28.4 | 29.6 | 24.75 | White |
| Hakkari-20 | 13.6 | 12.1 | 46.6 | 48.5 | 30.20 | Brown |
| Hakkari-23 | 42.5 | 41.2 | 44 | 45.8 | 43.38 | Beige |
| Hakkari-28 | 27.1 | 28.2 | 11.3 | 11.8 | 19.60 | Beige |
| Hakkari-31 | 39.2 | 40.7 | 32.3 | 33.6 | 36.45 | Dark red |
| Hakkari-37 | 39.5 | 41.1 | 55.3 | 57.5 | 48.35 | Purple |
| Hakkari-38 | 27 | 26.1 | 51.9 | 54 | 39.75 | Brown |
| Hakkari-39 | 5.4 | 5.2 | 4.8 | 4.9 | 5.08 | Purple |
| Hakkari-43 | 20.3 | 21.1 | 5 | 5.2 | 12.90 | White |
| Hakkari-44 | 4.5 | 4.7 | 4.1 | 4.3 | 4.40 | White |
| Hakkari-51 | 42.6 | 42.3 | 42.7 | 44.4 | 43.00 | White |
| Hakkari-55 | 7.6 | 7.9 | 5.9 | 6.1 | 6.88 | White |
| Hakkari-59 | 5.4 | 5.7 | 4.9 | 5 | 5.25 | White |
| Hakkari-63 | 36.3 | 37.7 | 28.7 | 29.9 | 33.15 | Brown |
| Hakkari-65 | 1.8 | 1.9 | 2.4 | 2.5 | 2.15 | White |
| Hakkari-69 | 7.7 | 8 | 5.9 | 6.1 | 6.93 | White |
| Hakkari-71 | 16.6 | 17.3 | 5.1 | 5.3 | 11.08 | Beige |
| Hakkari-76 | 42.8 | 44.5 | 41.1 | 42.8 | 42.80 | Beige |
| Tokat-83 | 41.4 | 40.1 | 33.7 | 35 | 37.55 | Dark red |
| Maras-92 | 4.6 | 4.8 | 6.6 | 6.8 | 5.70 | White |
| Bitlis-5 | 21.6 | 22.5 | 25.5 | 26.5 | 24.03 | Purple |
| Bitlis-14 | 4.9 | 5.1 | 6.1 | 6.4 | 5.63 | White |
| Bitlis-16 | 11.4 | 10.9 | 4.5 | 4.7 | 7.88 | White |
| Bitlis-22 | 42.4 | 44.1 | 64.3 | 66.8 | 54.40 | Beige |
| Bitlis-25 | 27.6 | 28.7 | 60.3 | 62.7 | 44.83 | White |
| Bitlis-35 | 35.9 | 36.3 | 13.9 | 14.5 | 25.15 | Purple |
| Bitlis-40 | 43.9 | 44.7 | 12.4 | 12.9 | 28.48 | Purple |
| Bitlis-46 | 8.8 | 9.1 | 9.7 | 10.1 | 9.43 | White |
| Bitlis-48 | 5.9 | 6.2 | 4.8 | 5 | 5.48 | White |
| Bitlis-53 | 5.1 | 5.1 | 5.5 | 5.7 | 5.35 | White |
| Bitlis-66 | 44.4 | 45.2 | 38.1 | 39.6 | 41.83 | Yellow |
| Bitlis-69 | 17.1 | 17.7 | 37.5 | 39 | 27.83 | Purple |
| Bitlis-76 | 41.2 | 40.9 | 42.7 | 44.4 | 42.30 | White |
| Bitlis-79 | 5.8 | 6.1 | 5 | 5.1 | 5.50 | Purple |
| Bitlis-81 | 36.3 | 37.8 | 37 | 38.4 | 37.38 | White |
| Bitlis-90 | 16.8 | 17.4 | 47.7 | 49.6 | 32.88 | White |
| Bitlis-94 | 17 | 17.7 | 48.1 | 50 | 33.20 | Purple |
| Bitlis-97 | 7.3 | 7.6 | 8.9 | 9.3 | 8.28 | White |
| Bitlis-103 | 41.8 | 40.4 | 38.2 | 39.7 | 40.03 | White |
| Bitlis-105 | 17.5 | 18.2 | 37.1 | 38.5 | 27.83 | Purple |
| Bitlis-111 | 46.5 | 48.4 | 37.5 | 39 | 42.85 | Beige |
| Bitlis-114 | 36 | 37.5 | 32.5 | 33.8 | 34.95 | White |
| Bitlis-115 | 8.9 | 9.2 | 3.7 | 3.9 | 6.43 | Brown |
| Bitlis-117 | 17 | 15.7 | 44.4 | 46.1 | 30.80 | White |
| Bitlis-118 | 5.8 | 6 | 5.1 | 5.3 | 5.55 | White |
| Bitlis-119 | 7.2 | 7.5 | 3.7 | 3.9 | 5.58 | White |
| Bitlis-120 | 5.3 | 5.5 | 6 | 6.3 | 5.78 | White |
| Bitlis-121 | 36.2 | 35.7 | 43.6 | 45.4 | 40.23 | White |
| Bitlis-124 | 49.2 | 51.2 | 37.4 | 38.9 | 44.18 | Dark red |
| Malatya-3 | 33.3 | 34.7 | 25.4 | 26.4 | 29.95 | Purple |
| Malatya-13 | 24.6 | 25.6 | 25.5 | 26.5 | 25.55 | Purple |
| Malatya-14 | 15.1 | 15.7 | 14.4 | 15 | 15.05 | Beige |
| Malatya-18 | 29.6 | 29.8 | 32.4 | 33.7 | 31.38 | Brown |
| Malatya-25 | 41.3 | 43 | 44.1 | 45.8 | 43.55 | Beige |
| Malatya-28 | 53.5 | 53.7 | 34.7 | 36.1 | 44.50 | Beige |
| Malatya-32 | 44 | 45.7 | 37.2 | 38.6 | 41.38 | Black |
| Malatya-33 | 17.7 | 15.4 | 3.4 | 3.6 | 10.03 | Purple |
| Malatya-45 | 49.7 | 49.6 | 43.3 | 45 | 46.90 | White |
| Malatya-50 | 29.5 | 30.7 | 45.1 | 46.9 | 38.05 | Brown |
| Malatya-51 | 5 | 5.2 | 3.9 | 4.1 | 4.55 | White |
| Malatya-52 | 58.2 | 61.6 | 58.9 | 61.3 | 60.00 | White |
| Malatya-59 | 7.2 | 7.5 | 8.2 | 8.5 | 7.85 | Beige |
| Malatya-71 | 27.3 | 28.4 | 38.4 | 40 | 33.53 | White |
| Tunceli-1 | 29.3 | 30.4 | 39.2 | 40.8 | 34.93 | Beige |
| Tunceli-5 | 29.8 | 31 | 30.1 | 31.3 | 30.55 | Beige |
| Tunceli-11 | 21.1 | 21.9 | 41.8 | 43.5 | 32.08 | Beige |
| Van-1 | 7 | 7.3 | 9.3 | 9.7 | 8.33 | White |
| Van-11 | 27 | 24.1 | 44.2 | 46 | 35.33 | White |
| Van-13 | 44.7 | 46.5 | 42.5 | 44.2 | 44.48 | Purple |
| Van-17 | 28 | 27.1 | 35.7 | 37.1 | 31.98 | Brown |
| Van-19 | 29.5 | 31.7 | 38.6 | 40.1 | 34.98 | Purple |
| Van-25 | 4 | 4.2 | 4.4 | 4.6 | 4.30 | Purple |
| Van-27 | 3.6 | 3.7 | 4.4 | 4.5 | 4.05 | White |
| Van-29 | 3.4 | 3.6 | 4.1 | 4.2 | 3.83 | White |
| Van-33 | 45.2 | 49 | 43.4 | 45.1 | 45.68 | Brown |
| Van-36 | 6.8 | 7.3 | 2.95 | 3.07 | 5.03 | Yellow |
| Van-42 | 4.1 | 4.2 | 5.6 | 5.8 | 4.93 | White |
| Van-47 | 3.8 | 3.8 | 5 | 5.2 | 4.45 | White |
| Van-51 | 4.2 | 4.3 | 4.7 | 4.9 | 4.53 | White |
| Van-59 | 7.6 | 7.9 | 11.8 | 12.2 | 9.88 | Yellow |
| Van-64 | 3.8 | 3.9 | 3 | 3.1 | 3.45 | White |
| Van-65 | 5 | 5.2 | 3.4 | 3.6 | 4.30 | White |
| Van-68 | 4.5 | 4.6 | 3.8 | 3.9 | 4.20 | Beige |
| Elazig-2 | 59.7 | 62 | 35.1 | 36.5 | 48.33 | White |
| Elazig-7 | 4.7 | 4.9 | 6 | 6.3 | 5.48 | Purple |
| Elazig-9 | 6 | 6.2 | 5.9 | 6.2 | 6.08 | White |
| Elazig-10 | 5.2 | 5.8 | 4.2 | 4.4 | 4.90 | White |
| Elazig-14 | 29.5 | 30.7 | 26.3 | 27.3 | 28.45 | White |
| Elazig16 | 5.5 | 5.7 | 5.6 | 5.8 | 5.65 | White |
| Elazig-25 | 12.1 | 13.6 | 4.4 | 4.6 | 8.68 | White |
| Elazig-27 | 6 | 6.4 | 6.8 | 7.1 | 6.58 | White |
| Elazig-29 | 4.9 | 5.3 | 4.6 | 4.8 | 4.90 | White |
| Elazig-30 | 4.7 | 4.9 | 3.9 | 4.1 | 4.40 | White |
| Elazig-34 | 5.2 | 5.4 | 6 | 6.2 | 5.70 | White |
| Elazig-36 | 4.7 | 4.9 | 4.9 | 5.1 | 4.90 | White |
| Elazig-39 | 27.7 | 27.4 | 51 | 53.1 | 39.80 | Beige |
| Mus-1 | 4.8 | 4.9 | 4.7 | 4.9 | 4.83 | White |
| Mus-2 | 41.1 | 40.2 | 42.5 | 44.2 | 42.00 | White |
| Mus-7 | 6.1 | 6.4 | 4.9 | 5.1 | 5.63 | White |
| Mus-10 | 5.2 | 5.4 | 5 | 5.1 | 5.18 | White |
| Mus-15 | 6.5 | 6.6 | 10.2 | 10.6 | 8.48 | White |
| Mus-18 | 29.5 | 30.7 | 29.3 | 30.5 | 30.00 | White |
| Mus-22 | 40.2 | 40.8 | 19.4 | 20.2 | 30.15 | Purple |
| Mus-27 | 54.1 | 53.3 | 50.9 | 53 | 52.83 | Beige |
| Mus-28 | 8.5 | 8.8 | 8 | 8.3 | 8.40 | Yellow |
| Mus-34 | 6.4 | 6.8 | 4.7 | 4.8 | 5.68 | White |
| Mus-39 | 40.1 | 39.7 | 36.4 | 37.8 | 38.50 | White |
| Mus-41 | 6 | 6.2 | 6 | 6.2 | 6.10 | Black |
| Mus-42 | 62 | 64.5 | 50.8 | 52.8 | 57.53 | White |
| Mus-43 | 50.5 | 52.5 | 46.6 | 48.4 | 49.50 | White |
| Mus-46 | 25 | 25.9 | 27.9 | 29 | 26.95 | Black |
| Mus-48 | 63.1 | 65.5 | 32.7 | 34 | 48.83 | Black |
| Mus-49 | 63.6 | 66.2 | 47.3 | 49.2 | 56.58 | Purple |
| Mus-50 | 32.8 | 34.4 | 36.7 | 38.1 | 35.50 | Dark red |
| Mus-51 | 32.9 | 34.2 | 35.9 | 37.4 | 35.10 | Beige |
| Mus-52 | 54.1 | 53.2 | 41.3 | 42.9 | 47.88 | Beige |
| Mus-53 | 27.6 | 27.7 | 50.9 | 53 | 39.80 | Yellow |
| Sivas-3 | 6.4 | 6.6 | 6.2 | 6.5 | 6.43 | White |
| Sivas-4 | 5.3 | 5.5 | 5.7 | 5.9 | 5.60 | White |
| Sivas-7 | 30.3 | 31.5 | 20.3 | 21.1 | 25.80 | Beige |
| Sivas-12 | 4.9 | 5.1 | 4.3 | 4.5 | 4.70 | Beige |
| Sivas-13 | 10.2 | 10.8 | 5.1 | 5.3 | 7.85 | White |
| Sivas-16 | 6 | 6.3 | 5.2 | 5.4 | 5.73 | White |
| Sivas-17 | 2.32 | 2.22 | 4.7 | 4.8 | 3.51 | White |
| Sivas-18 | 2.96 | 3.08 | 4.5 | 4.7 | 3.81 | Dark red |
| Sivas-44 | 7.2 | 7.5 | 24.6 | 25.6 | 16.23 | Dark red |
| Sivas-62 | 4.3 | 4.5 | 4.5 | 4.7 | 4.50 | White |
| Sivas-68 | 5.3 | 5.5 | 5.9 | 6.2 | 5.73 | White |
| Sivas-69 | 29 | 30.2 | 38.6 | 40.1 | 34.48 | White |
| Sivas-70 | 4.8 | 5 | 5.2 | 5.4 | 5.10 | Dark red |
| Bilecik-1 | 4.6 | 4.8 | 5 | 5.1 | 4.88 | White |
| Bilecik-2 | 19.8 | 20.6 | 6 | 6.2 | 13.15 | Brown |
| Bilecik-6 | 51.2 | 53.2 | 25.8 | 26.8 | 39.25 | White |
| Bilecik-7 | 8.8 | 9.2 | 7.2 | 7.5 | 8.18 | White |
| Bilecik-8 | 6.7 | 6.9 | 3.1 | 3.3 | 5.00 | White |
| Bilecik-10 | 4.8 | 5.03 | 6.9 | 7.2 | 5.98 | Purple |
| Balikesir-3 | 23.6 | 24.6 | 47.3 | 49.2 | 36.18 | Brown |
| Balikesir-4 | 4.8 | 5 | 5.6 | 5.8 | 5.30 | White |
| Balikesir-5 | 26.7 | 27.8 | 7 | 7.3 | 17.20 | Beige |
| Balikesir-6 | 4.6 | 4.8 | 5 | 5.2 | 4.90 | White |
| Balikesir-17 | 7.6 | 7.9 | 4.1 | 4.2 | 5.95 | White |
| Balikesir-18 | 5.7 | 5.9 | 5.4 | 5.6 | 5.65 | White |
| Balikesir-19 | 67.2 | 69.9 | 51.4 | 53.4 | 60.48 | Yellow |
| Balikesir-20 | 5.8 | 6 | 6.2 | 6.4 | 6.10 | Brown |
| Duzce-1 | 6.04 | 6.2 | 3.2 | 3.3 | 4.69 | Beige |
| Duzce-9 | 5.4 | 5.6 | 4.8 | 5 | 5.20 | Purple |
| Yalova-13 | 8.2 | 8.5 | 4.9 | 5.1 | 6.68 | White |
| Yalova-20 | 27.8 | 28.9 | 44.3 | 46 | 36.75 | White |
| Yalova-21 | 50.6 | 52.6 | 48 | 49.9 | 50.28 | Brown |
| Erzincan-1 | 5.6 | 5.8 | 4.3 | 4.5 | 5.05 | White |
| Erzincan-3 | 8.4 | 8.7 | 5 | 5.1 | 6.80 | White |
| Erzincan-4 | 3.5 | 3.6 | 7.2 | 7.5 | 5.45 | Dark red |
| Erzincan-5 | 7.3 | 7.6 | 5.1 | 5.3 | 6.33 | White |
| Bursa-1 | 5.5 | 5.7 | 4.9 | 5.1 | 5.30 | White |
| Bursa-22 | 9.4 | 9.8 | 4.3 | 4.5 | 7.00 | White |
| Nigde-Dermasyon | 7.6 | 7.9 | 2.84 | 2.96 | 5.33 | White |
| Nigde-Derinkuyu | 4.7 | 4.9 | 6.1 | 6.3 | 5.50 | White |
| Civiril-Bolu | 7.7 | 8 | 11 | 11.4 | 9.53 | White |
| Bolu-Goynuk | 6.8 | 7.1 | 4.6 | 4.8 | 5.83 | Beige |
| Moralaca | 57.7 | 60 | 47.9 | 49.8 | 53.85 | Dark red |
| Akman × | 8.4 | 8.8 | 11.5 | 11.9 | 10.15 | White |
| Goynuk × | 4.9 | 5.1 | 12.9 | 13.4 | 9.075 | White |
| Karacasehir × | 6.6 | 6.8 | 8.2 | 8.5 | 7.525 | White |
| Onceler× | 47.8 | 49.4 | 36.3 | 37.7 | 42.8 | Dark red |
| Göksun× | 7.9 | 8.2 | 16 | 16.6 | 12.175 | White |
| Addag× | 7.8 | 7.9 | 9.3 | 9.7 | 8.675 | White |

× Commercial cultivars
