# Supplementary material for: Uncovering Phenotypic Diversity and DArTseq Marker Loci Associated with Antioxidant Activity in Common Bean
Source: Genes (Basel). 2019 Dec 28;11(1):36. doi: 10.3390/genes11010036 (PMC7016922; doi:10.3390/genes11010036)
Supplement: Supplementary file 1 [file genes-11-00036-s001.zip › Table S3.docx]

**Table S3.** Comparison of mean, maximum and minimum for antioxidant activity (µmol TE/g fw) between Turkish common bean landraces and commercial cultivars under four environments

|  | Environments | Minimum | Maximum | *M±SD |
| --- | --- | --- | --- | --- |
| Landraces | Bolu 2017 | 1.87 | 67.2 | 19.78 ±17.7 |
|  | Bolu 2018 | 1.9 | 69.9 | 20.25±18.2 |
|  | Sivas 2017 | 2.4 | 64.3 | 19.65±18.2 |
|  | Sivas 2018 | 2.5 | 66.8 | 20.43±18.9 |
|  | Overall Mean | 2.15 | 60.4 | 20.03±16.2 |
| Cultivars | Bolu 2017 | 4.9 | 47.8 | 13.9±16.6 |
|  | Bolu 2018 | 5.1 | 49.4 | 14.3±17.2 |
|  | Sivas 2017 | 8.2 | 36.3 | 15.7±10.4 |
|  | Sivas 2018 | 8.5 | 37.7 | 16.3±10.8 |
|  | Overall Mean | 7.5 | 42.8 | 15.1±13.6 |

*M±SD: Mean and standard deviation
