# Supplementary material for: Uncovering Phenotypic Diversity and DArTseq Marker Loci Associated with Antioxidant Activity in Common Bean
Source: Genes (Basel). 2019 Dec 28;11(1):36. doi: 10.3390/genes11010036 (PMC7016922; doi:10.3390/genes11010036)
Supplement: Supplementary file 1 [file genes-11-00036-s001.zip › Table S1.docx]

Table S1. Passport data of Turkish common bean germplasm used in this study

| Accession Number | Names of Landraces | Collection Province | District | Village | Altitude (m) | Coordinates |
| --- | --- | --- | --- | --- | --- | --- |
| 1 | Bingol-1 | Bingol | Genç | Selvi Beldesi | 964 | 38° 34319/40° 18917 |
| 2 | Bingol-6 | Bingol | Ilıcalar | Merkez | 1161 | 38° 58893/40° 40699 |
| 3 | Bingol-7 | Bingol | Merkez | Alatepe | 1154 | 39° 03502/40° 45401 |
| 4 | Bingol-11 | Bingol | Merkez | Çobantaşı | 1542 | 39° 04033/40° 48557 |
| 5 | Bingol-16 | Bingol | Adaklı | Gökçeli | 1335 | 39° 12738/40° 25142 |
| 6 | Bingol-18 | Bingol | Kiğı | Güneyağıl | 1489 | 39° 17427/40° 20136 |
| 7 | Bingol-25 | Bingol | Solhan | Kavaklıdere | 1176 | 38° 55287/40° 56822 |
| 8 | Bingol-33 | Bingol | Yedisu | Şen Mezrası | - | - |
| 9 | Bingol-36 | Bingol | Yedisu | Muz | - | - |
| 10 | Bingol-44 | Bingol | Yedisu | Kürdan | - | - |
| 11 | Bingol-45 | Bingol | Yedisu | Kürdan | - | - |
| 12 | Bingol-52 | Bingol | Yedisu | Eski Balta | - | - |
| 13 | Bingol-53 | Bingol | Yedisu | Eski Balta | - | - |
| 14 | Bingol-58 | Bingol | Yedisu | Kara Polat | - | - |
| 15 | Bingol-60 | Bingol | Yedisu | Döşengi | - | - |
| 16 | Bingol-61 | Bingol | Yedisu | Kara Polat | - | - |
| 17 | Bingol-63 | Bingol | Yedisu | Güzgülü | - | - |
| 18 | Bingol-65 | Bingol | Karlıova | Üçevler | - | - |
| 19 | Hakkari-7 | Hakkari | Merkez | Otluca | 2054 | 37° 36246/43° 42370 |
| 20 | Hakkari-11 | Hakkari | Merkez | Üzümcü | 2097 | 37° 36332/43° 42526 |
| 21 | Hakkari-12 | Hakkari | Merkez | Üzümcü | 2097 | 37° 36332/43° 42526 |
| 22 | Hakkari-13 | Hakkari | Merkez | Ağaçdibi | 2097 | 37° 29370/43° 38184 |
| 23 | Hakkari-16 | Hakkari | Merkez | Çimenli | 1137 | 37° 29096/43° 37693 |
| 24 | Hakkari-20 | Hakkari | Merkez | Üzümcü | 1135 | 37° 29773/43° 34389 |
| 25 | Hakkari-23 | Hakkari | Merkez | Taşbaşı | 970 | 37° 23929/43° 29723 |
| 26 | Hakkari-28 | Hakkari | Çukurca | Narlı | 875 | 37° 16013/43° 35195 |
| 27 | Hakkari-31 | Hakkari | Merkez | Bay | 1832 | 37° 32687/43° 43333 |
| 28 | Hakkari-37 | Hakkari | Merkez | Merzan | 1993 | 37° 34095/43° 42308 |
| 29 | Hakkari-38 | Hakkari | Merkez | Merzan | 1993 | 37° 34095/43° 42308 |
| 30 | Hakkari-39 | Hakkari | Merkez | Merzan | 1993 | 37° 34095/43° 42308 |
| 31 | Hakkari-43 | Hakkari | Merkez | Durankaya | 1764 | 37° 33418/43° 37329 |
| 32 | Hakkari-44 | Hakkari | Merkez | Durankaya | 1764 | 37° 33418/43° 37329 |
| 33 | Hakkari-51 | Hakkari | Merkez | Rezan | 1601 | 37° 42104/43° 56276 |
| 34 | Hakkari-55 | Hakkari | Yüksekova | Bağışlı | 1811 | 37° 43279/44° 02206 |
| 35 | Hakkari-59 | Hakkâri | Yüksekova | Armutdüzü | 2090 m | 37º 40771 / 43º 57535 |
| 36 | Hakkari-63 | Hakkari | Yüksekova | Su Üstü | 1955 | 37° 35208/43° 04488 |
| 37 | Hakkari-65 | Hakkari | Yüksekova | Büyük Çiftlik | 1955 | 37° 35208/43° 04488 |
| 38 | Hakkari-69 | Hakkari | Yüksekova | Merkez | 1915 | 37° 32928/44° 08427 |
| 39 | Hakkari-71 | Hakkari | Şemdinli | Güzelkonak | 1724 | 37° 25223/44° 29056 |
| 40 | Hakkari-76 | Hakkari | Merkez | Üzümcü | 1135 | 37° 29773/43° 34389 |
| 41 | Tokat-83 | Tokat | - | - | - | - |
| 42 | Maras-92 | Kahramanmaraş | - | - | - | - |
| 43 | Bitlis-5 | Bitlis | Hizan | Merkez | 1629 | 38° 13424/42° 21614 |
| 44 | Bitlis-14 | Bitlis | Hizan | Akbıyık | 1522 | 38° 11967/42° 20644 |
| 45 | Bitlis-16 | Bitlis | Hizan | Yemişli | 1638 | 38° 12806/42° 21679 |
| 46 | Bitlis-22 | Bitlis | Hizan | Bahçelievler | 1521 | 38° 12806/42° 21679 |
| 47 | Bitlis-25 | Bitlis | Hizan | Kalkanlı | 2004 | 38° 07704/42° 37670 |
| 48 | Bitlis-35 | Bitlis | Hizan | Soğuksu | 1365 | 38° 06783/42° 33292 |
| 49 | Bitlis-40 | Bitlis | Hizan | Gayda | 1271 | 38° 10051/42° 22985 |
| 50 | Bitlis-46 | Bitlis | Tatvan | Yolalan | 1645 | 38° 16080/42° 18559 |
| 51 | Bitlis-48 | Bitlis | Merkez | Çınarbaşı | 1710 | 38° 15861/42° 17972 |
| 52 | Bitlis-53 | Bitlis | Merkez | Kuşlu | 1615 | 38° 19739/42° 14841 |
| 53 | Bitlis-66 | Bitlis | Mutki | Yumrumeşe | 1459 | 38° 26765/41° 51660 |
| 54 | Bitlis-69 | Bitlis | Mutki | Kavakbaşı | 1303 | 38° 28884/41° 48924 |
| 55 | Bitlis-71 | Bitlis | Mutki | Yenidoğan | 1197 | 38° 29116/41° 47168 |
| 56 | Bitlis-79 | Bitlis | Mutki | Eller | 1423 | 38° 28878/41° 43845 |
| 57 | Bitlis-81 | Bitlis | Güroymak | Yazlıkonak | 1810 | 38° 30257/42° 07150 |
| 58 | Bitlis-90 | Bitlis | Güroymak | Aşağıkolbaşı | 1655 | 38° 32695/42° 06804 |
| 59 | Bitlis-94 | Bitlis | Güroymak | Arpacık | 1700 | 38° 30930/42° 05787 |
| 60 | Bitlis-97 | Bitlis | Güroymak | Kuştaşı | 2002 | 38° 29645/42° 04575 |
| 61 | Bitlis-103 | Bitlis | Tatvan | Taşdemir | 1828 | 38° 27451/42° 23777 |
| 62 | Bitlis-105 | Bitlis | Tatvan | Çamaltı | 1728 | 38° 27483/42° 26602 |
| 63 | Bitlis-111 | Bitlis | Tatvan | Reşadiye | 1689 | 38° 29404/42° 32232 |
| 64 | Bitlis-114 | Bitlis | Merkez | Çınarbaşı | 1459 | 38° 26765/42° 51660 |
| 65 | Bitlis-115 | Bitlis | Mutki | Yumrumeşe | 2002 | 38° 29645/42° 04575 |
| 66 | Bitlis-117 | Bitlis | Merkez | Kuşlu | 1615 | 38° 19739/42° 14841 |
| 67 | Bitlis-118 | Bitlis | Tatvan | Kırkbulak | 1752 | 38° 24726/42° 16166 |
| 68 | Bitlis-119 | Bitlis | Hizan | Yemişli | 1638 | 38° 12806/42° 21679 |
| 69 | Bitlis-120 | Bitlis | Merkez | Yolalan | 1543 | 38° 17889/42° 15891 |
| 70 | Bitlis-121 | Bitlis | Mutki | Yumrumeşe | 1459 | 38° 26765/41° 51660 |
| 71 | Bitlis-124 | Bitlis | Güroymak | Yazlıkonak | 1615 | 38° 19739/42° 14841 |
| 72 | Malatya-3 | Malatya | Doğanşehir | Erkenek Bel. | 1388 | 37° 55785/37° 56501 |
| 73 | Malatya-13 | Malatya | Doğanşehir | Kurucaova Bel | 1369 | 37° 59707/38° 01503 |
| 74 | Malatya-14 | Malatya | Doğanşehir | Savaklı | 1364 | 38° 02576/37° 54593 |
| 75 | Malatya-18 | Malatya | Doğanşehir | Elmalı | 1410 | 38° 03339/37° 44688 |
| 76 | Malatya-25 | Malatya | Doğanşehir | Çığlık | 1235 | 38° 06477/37° 55440 |
| 77 | Malatya-28 | Malatya | Doğanşehir | Güroba | 1459 | 38° 05052/37° 57494 |
| 78 | Malatya-32 | Malatya | Doğanşehir | Çömlekoba | 1370 | 38° 05372/37° 56691 |
| 79 | Malatya-33 | Malatya | Doğanşehir | Polat Bel. | 1270 | 38° 09447/37° 51215 |
| 80 | Malatya-45 | Malatya | Akçadağ | Ören | 1158 | 38° 14905/37° 55605 |
| 81 | Malatya-50 | Malatya | Hekimhan | Çayevleri Mah. | 1457 | 38° 48854/37° 54964 |
| 82 | Malatya-51 | Malatya | Yeşilyurt | Aşağıköy | 1456 | 38° 09010/38° 18332 |
| 83 | Malatya-52 | Malatya | Doğanşehir | Merkez | 1280 | 38° 06477/37° 55440 |
| 84 | Malatya-59 | Malatya | Doğanşehir | Kurucaova | 1369 | 37° 59707/38° 01503 |
| 85 | Malatya-71 | Malatya | Doğanşehir | Güroba | 1465 | 38° 05052/37° 57494 |
| 86 | Tunceli-1 | Tunceli | Mazgirt | Merkez | 1122 | 39° 00014/39° 34766 |
| 87 | Tunceli-5 | Tunceli | Ovacık | Yeşilova | 1289 | 39° 20037/39° 05286 |
| 88 | Tunceli-11 | Tunceli | Pertek | Beydamı | - | - |
| 89 | Van-1 | Van | Gürpınar | Merkez | 1748 | 38° 19126/43° 22555 |
| 90 | Van-11 | Van | Çatak | Elmacı | 1807 | 38° 04867/43° 04475 |
| 91 | Van-13 | Van | Çatak | Bilgi | 1702 | 38° 05736/43° 15575 |
| 92 | Van-17 | Van | Çatak | Bilgi | 1702 | 38° 05736/43° 15575 |
| 93 | Van-19 | Van | Çatak | Alacayar | 1629 | 38° 01890/43° 08884 |
| 94 | Van-25 | Van | Çatak | Merkez | 1502 | 38° 00451/43° 03619 |
| 95 | Van-27 | Van | Çatak | Merkez | 1783 | 38° 00721/43° 04473 |
| 96 | Van-29 | Van | Başkale | Albayrak | 2072 m | 38º 08452 / 44º 12332 |
| 97 | Van-33 | Van | Başkale | Çaldıran | 2005 | 37° 47409/44° 07448 |
| 98 | Van-36 | Van | Başkale | Belliyurt | 1876 | 37° 49064/44° 06905 |
| 99 | Van-42 | Van | Erciş | Merkez | 1704 | 39° 01746/43° 21668 |
| 100 | Van-47 | Van | Erciş | Merkez | 1689 | 39° 00036/43° 21362 |
| 101 | Van-51 | Van | Başkale | Barış | 2244 | 38° 01147/43° 39146 |
| 102 | Van-64 | Van | Bahçesaray | Ünlüce | 1702 | 38° 31128/42° 19587 |
| 103 | Van-65 | Van | Bahçesaray | Ünlüce | 1702 | 38° 31128/42° 19587 |
| 104 | Van-68 | Van | Bahçesaray | Elmayaka | 1705 | 38° 30546/42° 19126 |
| 105 | Van-59 | Van | Çatak | Elmacı | 1807 | 38° 04867/43° 04475 |
| 106 | Elazig-2 | Elazig | Palu | Seydilli | 877 | 38° 41578/39° 53162 |
| 107 | Elazig-7 | Elazig | Palu | Gömeçbağlar | 956 | 38° 37887/39° 51625 |
| 108 | Elazig-9 | Elazig | Palu | Keklikdere | 870 | 38° 36885/39° 49865 |
| 109 | Elazig-10 | Elazig | Palu | Baltaşı | 919 | 38° 35361/39° 47344 |
| 110 | Elazig-14 | Elazig | Maden | Gezin | 919 | 38° 35361/39° 47344 |
| 111 | Elazig-16 | Elazig | Maden | Kızıltepe | 1291 | 38° 28865/39° 31155 |
| 112 | Elazig-25 | Elazig | Maden | Yıldızhan | 1313 | 38° 21174/39° 22660 |
| 113 | Elazig-27 | Elazig | Sivrice | Başkaynak | 1390 | 38° 22855/39° 22217 |
| 114 | Elazig-29 | Elazig | Sivrice | Elmasuyu | 1364 | 38° 24728/39° 23341 |
| 115 | Elazig-30 | Elazig | Maden | Gezin | 1350 | 38° 30760/39° 33182 |
| 116 | Elazig-34 | Elazig | Maden | Yeşilova | 1503 | 38° 32905/39° 33695 |
| 117 | Elazig-36 | Elazig | Maden | Küçükova | 1410 | 38° 32551/39° 32526 |
| 118 | Elazig-39 | Elazig | Maden | Gezin | 1350 | 38° 30760/39° 33182 |
| 119 | Mus-1 | MusMus | Malazgirt | Gülkuru | 1607 | 39° 05869/42° 38738 |
| 120 | Mus-2 | Mus | Bulanık | Güllüova | 1550 | 39° 03619/42° 19105 |
| 121 | Mus-7 | Mus | Bulanık | Güllüova | 1550 | 39° 03619/42° 19105 |
| 122 | Mus-10 | Mus | Bulanık | Balotu | 1489 | 39° 06752/42° 08046 |
| 123 | Mus-15 | Mus | Bulanık | Değirmensuyu | 1514 | 39° 10268/42° 05099 |
| 124 | Mus-18 | Mus | Korkut | Sazlıkbaşı | 1293 | 39° 40424/41° 58975 |
| 125 | Mus-22 | Mus | Hasköy | Merkez | 1315 | 38° 38175/41° 46056 |
| 126 | Mus-27 | Mus | Hasköy | Azıklı | 1369 | 38° 38595/41° 44016 |
| 127 | Mus-28 | Mus | Hasköy | Kültür | 1278 | 38° 40889/41° 41773 |
| 128 | Mus-34 | Mus | Merkez | Akpınar | 1400 | 39° 10591/41° 30486 |
| 129 | Mus-39 | Mus | Varto | Tepeköy | 1280 | 39° 05383/41° 30168 |
| 130 | Mus-41 | Mus | Varto | Tepeköy | 1280 | 39° 05383/41° 30168 |
| 131 | Mus-42 | Mus | Varto | Özenç | 1468 | 39° 06895/41° 30281 |
| 132 | Mus-43 | Mus | Varto | Taşçı | 1577 | 39° 12636/41° 23917 |
| 133 | Mus-46 | Mus | Bulanık | Güllüova | 1550 | 39° 03619/42° 19105 |
| 134 | Mus-48 | Mus | Bulanık | Güllüova | 1550 | 39° 03619/42° 19105 |
| 135 | Mus-49 | Mus | Bulanık | Güllüova | 1550 | 39° 03619/42° 19105 |
| 136 | Mus-50 | Mus | Bulanık | Balotu | 1489 | 39° 06752/42° 08046 |
| 137 | Mus-51 | Mus | Bulanık | Adıvar | 1463 | 38° 13447/42° 10513 |
| 138 | Mus-52 | Mus | Hasköy | Merkez | 1350 | 38° 13447/42° 10513 |
| 139 | Mus-53 | Mus | Hasköy | Azıklı | 1369 | 38° 38595/41° 44016 |
| 140 | Sivas-3 | Sivas | Suşehri | Arpacı | 1050 | 40° 957/38° 539 |
| 141 | Sivas-4 | Sivas | Suşehri | Günlüce | 1050 | 40° 957/38° 539 |
| 142 | Sivas-7 | Sivas | Suşehri | Akşar | 1050 | 40° 957/38° 539 |
| 143 | Sivas-12 | Sivas | Hafik | Yakaboyu | 1350 | 39° 510/37° 230 |
| 144 | Sivas-13 | Sivas | Kangal | Akpınar | 1540 | 39° 130/37° 240 |
| 145 | Sivas-16 | Sivas | Divriği | Arıkbaşı | 1250 | 39° 240/38° 70 |
| 146 | Sivas-17 | Sivas | İmranlı | Başlıca | 1650 | 39° 5248/38° 758 |
| 147 | Sivas-18 | Sivas | İmranlı | Gökdere | 1650 | 39° 5248/38° 758 |
| 148 | Sivas44 | - | - | - | - | - |
| 149 | Sivas62 | - | - | - | - | - |
| 150 | Sivas68 | - | - | - | - | - |
| 151 | Sivas69 | Sivas | - | - | - | - |
| 152 | Sivas-70 | Sivas | - | - | - | - |
| 153 | Bilecik-1 | Bilecik | Pazaryeri | Dereköy | 786 | 39° 59′38″/29° 54′41″ |
| 154 | Bilecik-2 | Bilecik | Pazaryeri | Günyurdu | 805 | 40° 0′5.9″/29° 54′9″ |
| 155 | Bilecik-6 | Bilecik | Pazaryeri | Dereköy | 876 | 39° 59′38″/29° 54′41″ |
| 156 | Bilecik-7 | Bilecik | Pazaryeri | Dereköy | 876 | 39° 59′38″/29° 54′41″ |
| 157 | Bilecik-8 | Bilecik | Pazaryeri | Dereköy | 876 m | 39° 59’ 38’ / 29° 54’ 41’’ |
| 158 | Bilecik-10 | Bilecik | Pazaryeri | Dereköy | 876 | 39° 59′38″/29° 54′41″ |
| 159 | Balikesir-3 | Balikesir | Manyas | Salur Mah. | 29 | 40° 05′51″/27° 56′11″ |
| 160 | Balikesir-4 | Balikesir | Manyas | Akçaova Mah. | 30 | 40° 07′16”/27° 51′18″ |
| 161 | Balikesir-5 | Balikesir | İvrindi | Ayaklı Köyü | 404 | 39.516°/27.364° |
| 162 | Balikesir-6 | Balikesir | İvrindi | Ayaklı Köyü | 403 | 39.516°/27.364° |
| 163 | Balikesir-17 | Balikesir | Sındırgı | Kürendere | 1051 | 39.313°/28.571° |
| 164 | Balikesir-18 | Balikesir | Sındırgı | Kürendere | 1051 | 39.313°/28.571° |
| 165 | Balikesir-19 | Balikesir | Sındırgı | Kürendere | 1051 | 39.313°/28.571° |
| 166 | Balikesir-20 | Balikesir | Sındırgı | Kürendere | 1051 | 39.313°/28.571° |
| 167 | Duzce-1 | Duzce | Merkez | Derdin | 859 | 40.711°/31.228° |
| 168 | Duzce-9 | Duzce | Merkez | Darıca Mah. | 163 | 40° 49′18′′/31° 10′26″ |
| 169 | Yalova-13 | Yalova | Çiftlikköy | Kabaklı | 125 | 40° 39′30″/29° 24′36″ |
| 170 | Yalova-20 | Yalova | Çınarcık | Ortaburun | 689 | 40° 37′04″/29° 09′00″ |
| 171 | Yalova-21 | Yalova | Çınarcık | Ortaburun | 688 | 40° 37′04″/29° 09′00″ |
| 172 | Erzincan-1 | Erzincan | Refahiye | Merkez | 1589 | 39° 544/38° 467 |
| 173 | Erzincan-3 | Erzincan | Kemah | Gökkaya | 1130 | 39° 3610/39° 28 |
| 174 | Erzincan-4 | Erzincan | Kemaliye | Merkez | 950 | 39° 1539/38° 2948 |
| 175 | Erzincan-5 | Erzincan | Kemaliye | Akçalı | 950 | 39° 1539/38° 2948 |
| 176 | Bursa-1 | Bursa | Yenişehir | Fethiye | 335 | 40.289°/29.445° |
| 177 | Bursa-22 | Bursa | Kestel | Aksu | 360 | 40.169°/29.317° |
| 178 | Dermasyon | Nigde | - | - | - | - |
| 179 | Derinkiyu | Nigde | - | - | - | - |
| 180 | Civril-Bolu | Bolu | Merkez | Doğancı Mah. | 842 | 40° 40′45″/31° 33′30″ |
| 181 | Bolu-Goynuik | Bolu | Merkez | Doğancı Mah. | 842 m | 40°40′45″ / 31°33′30″ |
| 182 | Moralaca | Bolu | Merkez | Doğancı Mah. | 842 m | 40°40′45″ / 31°33′30″ |
| 183 | Akman × |  |  |  |  |  |
| 184 | Goynuk × |  |  |  |  |  |
| 185 | Karacasehir × |  |  |  |  |  |
| 186 | Onceler× |  |  |  |  |  |
| 187 | Göksun× |  |  |  |  |  |
| 188 | Addag× |  |  |  |  |  |

× Commercial cultivars
