# Supplementary figures and images for: Uncovering Phenotypic Diversity and DArTseq Marker Loci Associated with Antioxidant Activity in Common Bean
Source: Genes (Basel). 2019 Dec 28;11(1):36. doi: 10.3390/genes11010036 (PMC7016922; doi:10.3390/genes11010036)

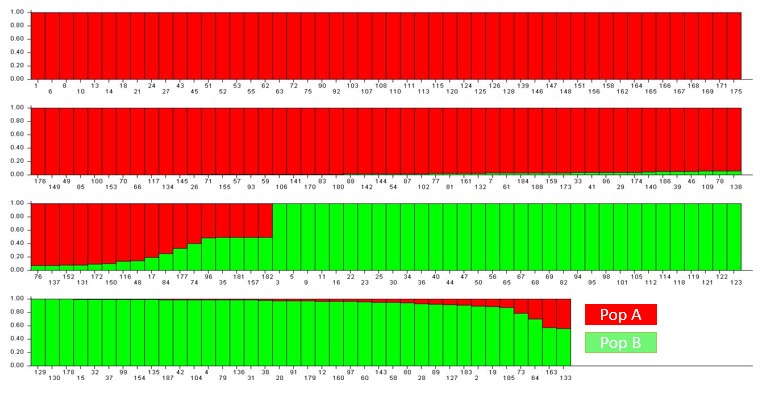

Supplement: Supplementary file 1 [file genes-11-00036-s001.zip › Figure S1.jpg]

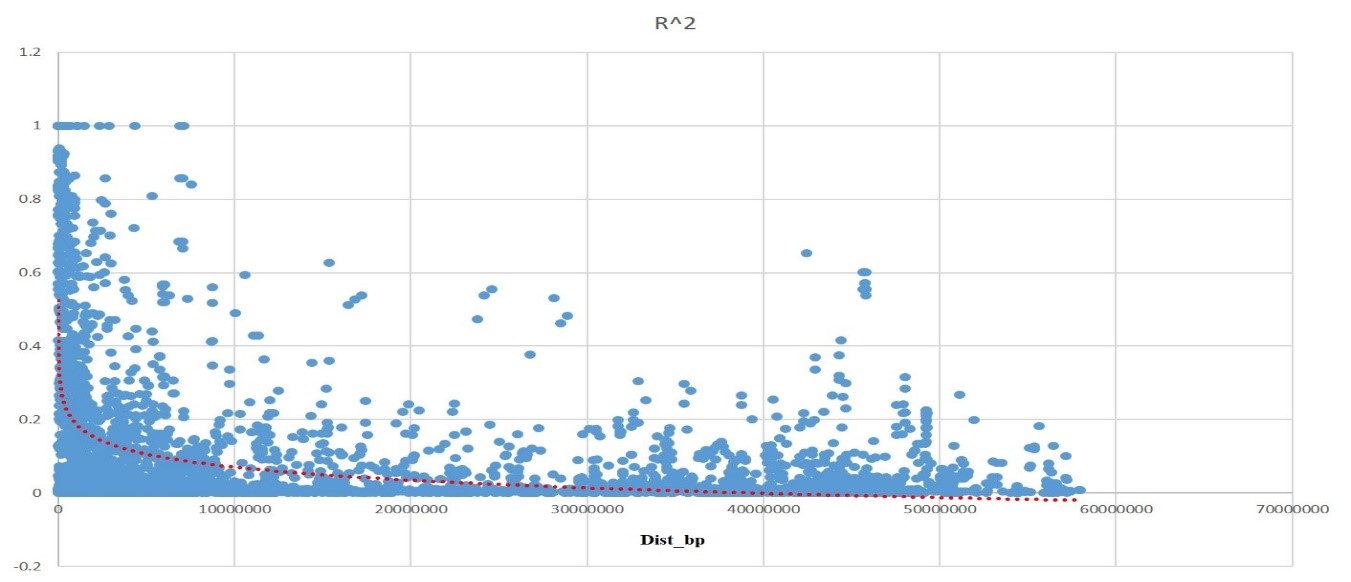

Supplement: Supplementary file 1 [file genes-11-00036-s001.zip › Figure S2.jpg]

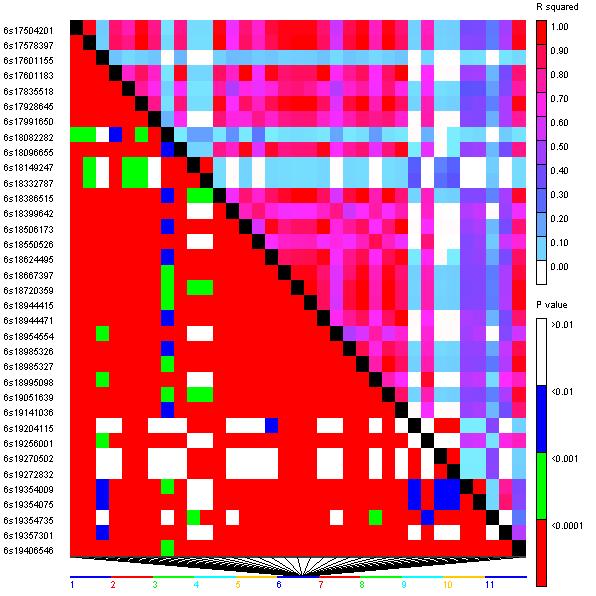

Supplement: Supplementary file 1 [file genes-11-00036-s001.zip › Figure S3.jpg]
